# Microenvironmental Drivers of Glioma Progression

**DOI:** 10.3390/ijms26052108

**Published:** 2025-02-27

**Authors:** Hyun Ji Jang, Jong-Whi Park

**Affiliations:** 1 Department of Life Sciences, College of BioNano Technology, Gachon University, Seongnam 13120, Republic of Korea; jangcindy@naver.com; 2 Department of Health Sciences and Technology, GAIHST, Gachon University, Incheon 21999, Republic of Korea

**Keywords:** glioma, tumor microenvironment, immune modulation, neuronal interactions, extracellular matrix

## Abstract

Gliomas, particularly glioblastoma (GBM), are among the most challenging brain tumors due to their complex and dynamic tumor microenvironment (TME). The TME plays a pivotal role in tumor progression, immune evasion, and resistance to therapy through intricate interactions among glioma cells, immune components, neurons, astrocytes, the extracellular matrix, and the blood-brain barrier. Targeting the TME has demonstrated potential, with immunotherapies such as checkpoint inhibitors and neoadjuvant therapies enhancing immune responses. Nonetheless, overcoming the immunosuppressive landscape and metabolic adaptations continues to pose significant challenges. This review explores the diverse cellular and molecular mechanisms that shape the glioma TME. A deeper understanding of these mechanisms holds promise for providing novel therapeutic opportunities to improve glioma treatment outcomes.

## 1. Introduction

The tumor microenvironment (TME) is a dynamic and integral component of solid tumors, comprising not only malignant cells but also various supportive elements such as stromal cells, fibroblasts, immune cells, and components like the extracellular matrix (ECM) [1]. Tumor initiation often depends on a supportive microenvironment, as mutant cells can actively influence and remodel their surroundings to favor growth and survival. Additionally, signaling molecules such as growth factors, cytokines, and chemokines foster a tumor-permissive niche, facilitating growth, immune evasion, resistance to therapy, and metastasis. As tumors evolve, the bidirectional interactions between neoplastic cells and stromal components become increasingly complex, driven by ongoing signaling exchanges [2].

Gliomas are primary brain tumors that are classified based on their histological features and molecular characteristics, as defined by the World Health Organization (WHO) classification system [3]. Reflecting the tumor’s level of aggressiveness and malignancy, grades range from slow-growing, low-grade gliomas to highly aggressive forms such as glioblastoma (GBM), the most severe and rapidly progressing subtype. Despite the standard-of-care treatment comprising maximal surgical resection, temozolomide (TMZ) chemotherapy, and radiotherapy, the five-year survival rate remains at only 6%, with a median overall survival of approximately 15 months, urgently underscoring the need for more effective therapies to enhance patient outcomes [4,5].

**Figure 1:**
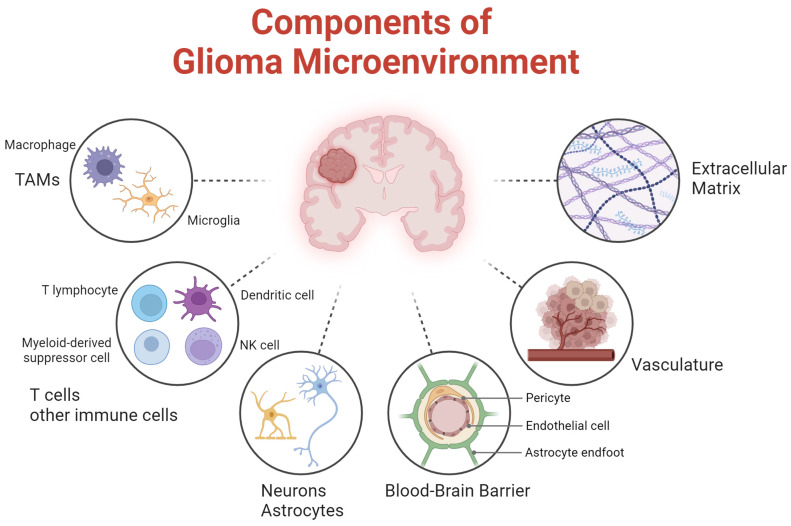
Schematic representation of the glioma microenvironment components. Key elements include immune components such as tumor-associated myeloid cells (TAMs), T lymphocytes and dendritic cells, and neural components including astrocytes and neurons. The extracellular matrix provides structural support, while the vasculature and blood-brain barrier, composed of endothelial cells, pericytes, and astrocyte endfeet, regulate nutrient exchange and immune infiltration.

The brain TME is unique relative to other cancers due to the presence of specialized, tissue-resident cells such as microglia, astrocytes, and neurons, which interact with tumor cells [6,7,8] (Figure 1). Additionally, the blood-brain barrier (BBB) or its tumor-altered form, the blood-tumor barrier (BTB), limits the entry of immune cells and therapeutic agents into the brain, presenting a significant challenge for effective immune response and drug delivery in brain cancers [9]. Governed by tightly interconnected endothelial cells, pericytes, and astrocytic endfeet, this selective permeability therefore suggests that targeting the microenvironment could offer potential therapeutic strategies to modulate these interactions and possibly improve outcomes for patients with gliomas [10,11]. The following section will explore the glioma microenvironment in detail, examining its variations across different glioma subtypes.

## 2. Glioma Progression and Microenvironment

Glioma progression can be driven by molecular and cellular changes, including microenvironmental factors such as hypoxia, immune suppression, and neuronal activity. The glioma microenvironment, a dynamic and complex ecosystem, surrounds glioma cells within the brain. It consists of various cellular and non-cellular elements that interact with tumor cells, thereby influencing their growth, invasion, and resistance to therapies. For instance, tumor-associated myeloid cells (TAMs) play a pivotal role in glioblastoma progression by facilitating creatine biosynthesis and transfer within hypoxic tumor niches [12]. Notably, SLC6A8-mediated creatine uptake supports glioblastoma cell survival and stemness under metabolic stress [12]. Moreover, myeloid-specific deletion of KDM6B in murine models has demonstrated enhanced proinflammatory responses, reduced tumor burden, and improved survival, highlighting the therapeutic potential of reprogramming TAMs [13].

GBM cell plasticity, driven by genetic mutations and microenvironmental factors, contributes to tumor heterogeneity and complicates treatment [14]. A notable example is the mesenchymal subtype transition, frequently observed as an evolutionary pathway in recurrent glioblastomas, potentially driven by microenvironmental pressures such as hypoxia, inflammation, and immune cell interactions [15,16]. While genetic or pharmacological targeting of IL-1β successfully reduced monocyte recruitment and improved survival in proneural GBM models, this approach was ineffective in mesenchymal subtypes due to the presence of constitutively active NF-κB signaling, underscoring the need for subtype-specific therapeutic strategies [17]. EGFRvIII, a common mutation in GBM, arises from a deletion of exons 2–7 in the EGFR gene, producing a constitutively active receptor that drives oncogenic signaling without ligand binding [18]. Beyond promoting glioma cell proliferation, this mutation significantly influences tumor-microenvironment interactions. Specifically, EGFRvIII alters the composition of extracellular vesicles, enriching them with pro-invasive proteins (CD44, CD151, ITGA6) while downregulating exosomal markers (CD81, CD82) [19].

Isocitrate dehydrogenase (IDH) mutation is a defining molecular characteristic of a subset of gliomas, particularly low-grade gliomas, including astrocytoma and oligodendroglioma. Both glioma subtypes demonstrate a shared developmental hierarchy comprising astrocytic, oligodendrocytic, and stem-like proliferative cells [20]. With increasing tumor grade, there is a significant expansion of malignant cell populations, accompanied by an increase in undifferentiated glioma cells, which contributes to tumor progression and therapeutic resistance [20]. The variation in gene expression profiles between IDH-mutant astrocytomas and oligodendrogliomas is primarily influenced by the tumor microenvironment and specific genetic mutations, rather than by distinct glial lineages. Notably, distinct immune interactions specific to each subtype are evident through varying TAM profiles in IDH-mutant gliomas, with astrocytomas showing a predominance of inflammatory TAMs [21]. Conversely, oligodendrogliomas are distinguished by a population enriched in ribosomal content exhibiting stem-like properties, as identified by Blanco-Carmona et al. [21].

IDH mutations further induce a reprogramming of myeloid cells toward an immature, immunosuppressive phenotype, which diminishes antigen presentation and facilitates tumor immune evasion. For instance, tryptophan metabolism through the kynurenine pathway promotes immunosuppressive myeloid cell states in IDH-mutant gliomas, contributing to the circumvention of immune responses [22]. In syngeneic mouse models, IDH1 mutations in gliomas downregulate genes related to chemotaxis, thereby reducing immune infiltration, including that of microglia and neutrophils, which may enhance patient survival [23].

Microglia survival is critically dependent on CSF-1R signaling, as its inhibition results in over 99% depletion in adult brains [24]. Lineage-tracing approaches have revealed that in the adult mouse brain, repopulated microglia derive exclusively from the rapid proliferation of a few surviving microglia following acute depletion, not from peripheral precursors [25]. Microglia, which are the resident immune cells of the brain, are more prevalent in IDH mutant gliomas. These tumors typically exhibit an immunosuppressive environment with lower levels of T cells and neutrophils, resulting in an “immunologically cold” landscape. By contrast, IDH wild-type gliomas and brain metastases exhibit a more heterogeneous immune landscape that includes a significant presence of monocyte-derived macrophages, neutrophils, and adaptive immune cells, leading to more complex immune interactions [26,27]. Given these differences in immune composition, it is crucial to identify reliable markers to distinguish between key myeloid populations to understand their roles in glioma progression. CD49D/Itga4 has been identified as a reliable marker for distinguishing bone marrow-derived macrophages from tumor-associated microglia [28]. Moreover, these two myeloid populations in brain tumors exhibit distinct transcriptional profiles influenced by pre-tumor epigenetic landscapes and tumor-driven reprogramming [28].

H3.3K27M mutations in diffuse midline gliomas are strongly associated with increased TAM infiltration, heightened immune suppression, and reduced survival compared to other histone variants [29]. Genetic and pharmacological interventions targeting chemokine receptors such as CCR1 and CCR5 have demonstrated efficacy in disrupting macrophage recruitment and enhancing anti-tumor immune activity, ultimately extending survival in murine glioma models [29].

## 3. Immune Cell Components of the Glioma Microenvironment

### 3.1. Microglia and Macrophages

Glioma progression is strongly influenced by tumor-associated microglia and bone marrow-derived macrophages, which have distinct localizations and functions that crucially shape the tumor microenvironment. Bone marrow-derived macrophages are predominantly localized to perivascular regions within the tumor parenchyma, while brain-resident microglia are primarily found in the peritumoral areas outside the tumor core [30,31]. Notably, higher levels of CD68-, CD163-, and CD206-positive glioma-associated microglia and macrophages correlate with prolonged survival in IDH1 R132H-non-mutant glioblastoma patients, suggesting that modulation of these immune populations could have therapeutic benefits [32].

Tumor-associated microglia facilitate glioma invasion by modulating the tumor microenvironment through the release of cytokines, growth factors, and extracellular matrix-degrading enzymes, thereby enhancing tumor proliferation [33]. For instance, Nf1 heterozygous microglia promote the proliferation of Nf1-deficient astrocytes via the secretion of hyaluronidase, a critical factor in tumor progression [34]. Similarly, Solga et al. identified Ccl5 as a crucial factor derived from microglia that promotes the growth of NF1-associated low-grade gliomas. Consistent with this finding, inactivating microglia with minocycline significantly reduces Ccl5 expression, thereby inhibiting glioma growth in vivo [35].

The phenotypic adaptation of glioblastoma-associated microglia into diverse functional states, such as phagocytic and dendritic cell-like roles, highlights the complexity of tumor-immune interactions [36]. Central to these adaptations is the spatial interaction between microglial CD39 and tumor-expressed CD73, which fosters an adenosine-enriched microenvironment in glioblastoma, facilitating immune evasion and poor outcomes [37]. Similarly, TFPI2 secretion has been demonstrated to actively recruit and polarize microglia towards an immunosuppressive phenotype through activation of the CD51-STAT6 signaling pathway [38]. The CX3C chemokine receptor 1 (CX3CR1) is a G-protein coupled receptor that binds its ligand CX3CL1 (fractalkine) and is primarily expressed on microglia, monocytes, macrophages, natural killer cells, and specific subsets of T cells. Lowering expression of CX3CR1 in microglia significantly postponed optic glioma development in a mouse model of neurofibromatosis type 1 [39].

Glioblastoma stem cells (GSCs) secrete periostin (POSTN), which acts as a chemoattractant for circulating monocytes that infiltrate the tumor and differentiate into M2-like macrophages via integrin αvβ3 signaling. These M2 macrophages establish an immunosuppressive niche, promoting glioblastoma progression [40]. Moreover, Kynurenine, produced by glioblastomas, activates the aryl hydrocarbon receptor (AHR) in macrophages, resulting in the induction of CD39 expression and enhanced adenosine production, which suppresses CD8+ T cell immunity and promotes GBM progression [41]. A recent pivotal study demonstrated that TREM2 blockade directs monocyte-to-macrophage differentiation towards pro-inflammatory states, laying the groundwork for innovative immunotherapy approaches in glioblastoma [42].

CSF-1R inhibition using either BLZ945 or PLX3397 leads to tumor regression and enhanced survival in PDGF-driven proneural GBM mouse models by modifying macrophage polarization rather than depleting these cells [43,44]. Interestingly, glioma-secreted factors such as GM-CSF and IFN-gamma support macrophage survival in the presence of the CSF-1R inhibitor, although M2 macrophage polarization is significantly diminished [43]. Additionally, macrophage-derived IGF-1 activates IGF-1R and PI3K signaling pathways in tumor cells, which promote tumor recurrence following CSF-1R inhibition [45]. Consistent with these observations, combining IGF-1R or PI3K inhibition with CSF-1R blockade markedly improves therapeutic outcomes and prolongs survival in preclinical GBM models [45].

Lipid-laden macrophages (LLMs) in glioblastoma acquire a pro-tumorigenic phenotype through the phagocytosis of cholesterol-rich myelin debris, thereby fueling MES-like tumor cell proliferation [46]. Research has shown that targeting the CD36 and ABCA1-mediated lipid transport disrupts tumor-promoting interactions between LLMs and glioblastoma cells [46]. In line with this evidence, glioblastoma-associated foam cells (TAFs) are generated from lipid droplet accumulation in macrophages, induced by tumor-derived extracellular vesicles under hypoxic conditions [47]. Moreover, the study underscored the therapeutic potential of DGAT1 and ACSL inhibitors in inhibiting lipid droplet formation and reducing TAF-mediated pro-tumorigenic activities, underscoring the pivotal role of lipid metabolism in the progression of glioblastoma [47].

### 3.2. T Cells and Other Immune Cells

The immunosuppressive TME influences brain tumor progression with distinct immune compositions. Notably, tissue-resident phagocytes are prevalent in the glioma TME, whereas tissue-invading leukocytes predominate in brain metastases (BrM), reflecting tumor type-dependent leukocyte diversity within the tumor microenvironment [48]. In pediatric high-grade gliomas, hypermutator and PXA-like tumors exhibit significant CD8+ T cell infiltration and associated improved survival with bevacizumab treatment, whereas histone H3-mutant tumors remain largely immune cold and are non-responsive to bevacizumab [49].

Single-cell RNA sequencing has revealed that glioma-infiltrating T cells co-express cytotoxic markers and NK cell genes, suggesting the presence of hybrid T cell states with distinctive functional properties [50]. These T cells express CD161, an inhibitory receptor that reduces their cytotoxic potential through interaction with CLEC2D on tumor and myeloid cells. Notably, genetic or antibody-mediated blockade of CD161 significantly enhances T cell-mediated killing of glioma cells [50]. Furthermore, tumor-derived Fibrinogen-like protein 2 (FGL2) suppresses the differentiation of CD103+ dendritic cells, thereby impairing CD8+ T cell-mediated immune responses against glioblastoma [51].

Lad et al. discovered that glioblastoma recruits immature neutrophils from the skull bone marrow, which differentiate into antigen-presenting hybrid neutrophils capable of activating T cells [52]. The study identified a unique dendritic-like tumor-associated neutrophil phenotype that suppresses glioblastoma growth through MHCII-dependent T cell activation mechanisms [52]. The compensatory influx of neutrophils following deletion of monocyte chemoattractant proteins promotes a proneural-to-mesenchymal shift in glioblastoma, a transition driven by neutrophil-derived TNF-α. This observation suggests that dual targeting of monocyte and neutrophil populations may be essential to prevent resistance and improve therapeutic outcomes in glioblastoma [16].

Myeloid-Derived Suppressor Cells (MDSCs) infiltrate both human and murine GBM tumors [53,54]. Glioblastoma-derived factors such as CCL20 and OPG elicit CCL2 production from macrophages and microglia, which, in turn, drives Treg and monocytic MDSC recruitment through CCR4 and CCR2 pathways [54]. Importantly, treatment with sunitinib significantly reduces MDSC counts, restores T cell proliferation and IFN-γ production, and enhances tumor immune infiltration [53].

## 4. Non-Immune Cell Components of the Glioma Microenvironment

### 4.1. Neurons

**Figure 2:**
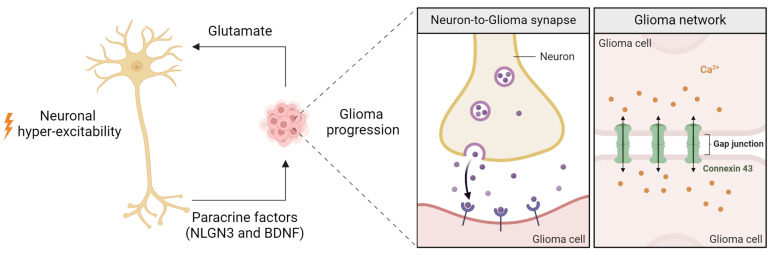
Neuron-Glioma interactions and tumor progression. Neuronal hyper-excitability and glioma progression are reciprocally linked. Neurons promote glioma growth through paracrine factors like NLGN3 and BDNF, while glioma cells enhance neuronal activity by releasing glutamate. Glioma cells also form neuron-to-glioma synapses, facilitating excitatory signaling, and establish an interconnected network via Connexin 43 gap junctions, enabling calcium signaling and tumor propagation.

Gliomas form synapse interactions with surrounding neurons, leveraging neuronal signals to support their own growth and facilitate invasion into neighboring brain tissues. Employing an orthotopic xenograft model of high-grade glioma, Venkatesh et al. demonstrated that neuronal activity promotes the secretion of synaptic protein neuroligin-3 (NLGN3), which in turn stimulates glioma growth [55] (Figure 2). Notably, NLGN3 induces a feedforward loop that further enhances its own expression in glioma cells [55]. In an activity-dependent manner, full-length NLGN3 is cleaved by ADAM10 (a disintegrin and metalloproteinase domain-containing protein 10), releasing microenvironmental NLGN3. This process is crucial for maintaining glioma proliferation in an orthotopic xenograft model, underscoring the therapeutic potential of ADAM10 inhibitors in curbing tumor growth [56].

Complementing these findings, single-cell RNA sequencing of human gliomas has revealed that a specific subset of glioma cells robustly expresses postsynaptic genes, including those encoding glutamate receptors of the AMPA subtype [57]. Moreover, subsequent ultrastructural and electrophysiological approaches have confirmed the functionality of direct glutamatergic synapses between presynaptic neurons and postsynaptic glioma cells. Consistent with this, treatment with an AMPA receptor antagonist or the forced expression of a dominant-negative form of GLUA2 (GLUA2-DN) significantly reduced glioma cell proliferation in vivo [57,58]. Furthermore, neuronal activity promotes the expansion of neurite-like tumor microtubes, consequently enhancing tumor cell networks [59] (Figure 2). Confocal time-lapse imaging was employed in this study to elucidate the dynamic interplay between glioma cells and neuronal activity, shedding light on mechanisms of invasion [59].

Recent research has further linked neuronal activity to glioma progression through BDNF-TrkB signaling, which enhances synaptic strength and AMPAR trafficking, thereby driving tumor growth [60]. Furthermore, radiotherapy has been shown to increase neuron-tumor network integration; however, when combined with AMPA receptor inhibition, there is a significant reduction in tumor progression [61]. Corroborating these findings, rabies virus tracing has demonstrated extensive glioma-neuron synaptic integration, predominantly involving glutamatergic neurons, with regional variation observable in the distribution of glioma-innervating neurons [61,62]. Interestingly, the secreted synaptogenic protein thrombospondin-1 (TSP-1) has been identified as enhancing intra-tumoral functional connectivity and promoting neuronal activity-dependent glioma proliferation [63].

Epileptic seizures in patients with brain tumors arise from glioma-induced neuronal activity. Sulfasalazine (Azulfidine), an inhibitor of the cystine/glutamate antiporter (xCT), has been shown to reduce epileptic activity in glioma-bearing mice [64]. Furthermore, elevated xCT/SLC7A11 expression correlates with increased extracellular glutamate, neuronal toxicity, and reduced overall survival in glioma patients [65,66]. Additionally, the expression of xCT/SLC7A11 in patient glioma tissue correlates with the peritumoral glutamate response to sulfasalazine, suggesting its potential as a predictive biomarker for the efficacy of sulfasalazine in modulating glutamate release [65]. Beyond increased SLC7A11 expression, a reduction in peritumoral GABAergic inhibition contributes to the development of a hyperexcitable neuronal network prone to seizures [67]. Additionally, Glypican 3 (GPC3) is upregulated in tumors expressing specific PIK3CA mutations, and its overexpression induces aberrant synapse formation, exacerbating network hyperexcitability and accelerating the onset of seizures within the tumor microenvironment [68]. D-2-hydroxyglutarate, a metabolite released by IDH-mutant gliomas, has been demonstrated to enhance the electrical activity of cultured rat cortical neurons in an NMDA receptor-dependent manner, further implicating tumor metabolism in neuronal dysfunction [69].

### 4.2. Astrocytes

**Figure 3:**
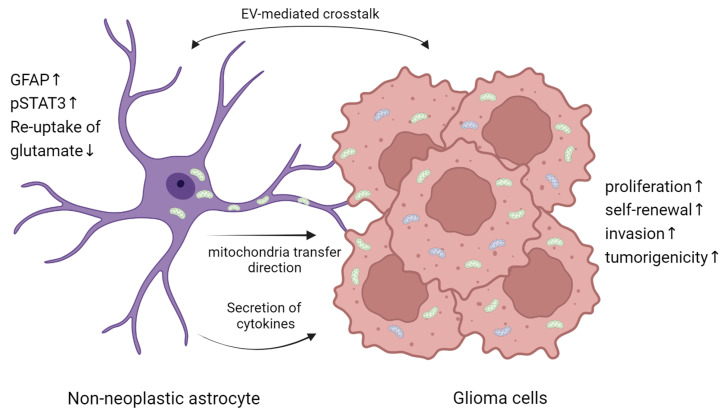
Astrocyte-Glioma Crosstalk in Tumor Progression. Non-neoplastic astrocytes interact with glioma cells through extracellular vesicle (EV)-mediated crosstalk, mitochondrial transfer, and cytokine secretion, including CCL2, CCL20, and TGF-β. Reactive astrocytes exhibit increased GFAP and STAT3 activation while reducing glutamate re-uptake, contributing to a tumor-supportive environment [74]. These interactions enhance glioma cell proliferation, self-renewal, invasion, and tumorigenicity.

The analogous expression profiles identified between mouse astrocyte subpopulations and the classical, mesenchymal, and neural subtypes of human GBM, but not with the proneural subtype [70]. Additionally, the emergence of specific astrocyte subpopulations correlates with key aspects of tumor progression, such as invasion and the onset of seizures [70]. This process is marked by astrocyte reactivity and scar formation, characterized by upregulation of GFAP, cellular hypertrophy, and pSTAT3 activation in peritumoral astrocytes [71]. Consequently, reactive astrogliosis leads to impaired potassium and glutamate uptake, largely due to decreased Kir4.1 expression, ultimately resulting in neuronal hyperexcitability and tumor-associated epilepsy [71]. Priego et al. reported that a subpopulation of reactive astrocytes with STAT3 activation can actively reprogram the brain microenvironment, transitioning it from a naïve to a pro-metastatic state [72]. High levels of phospho-STAT3 in reactive astrocytes are furthermore associated with decreased survival in patients with intracranial metastases, highlighting its clinical significance. Consistent with this, pharmacological inhibition of STAT3 signaling within the microenvironment significantly reduces the burden of brain metastasis [72]. Intriguingly, astrocytes actively contribute to glioblastoma progression through metabolic crosstalk, including direct transfer of intact mitochondria to tumor cells. This process, mediated through GAP43-dependent intercellular structures, enhances the bioenergetic capacity of glioblastoma cells, promoting their proliferation and tumorigenicity [73] (Figure 3).

### 4.3. The BBB and Vasculature

The BBB permits the passage of small hydrophilic molecules through transporters and small lipophilic molecules via passive diffusion, while it facilitates the crossing of larger or hydrophilic molecules through receptor-mediated transport, thereby maintaining brain homeostasis by ensuring the delivery of nutrients and the exclusion of toxins [75]. However, this rigorously controlled system is compromised under pathological conditions. As glioma progresses, it compromises the BBB and triggers inflammation, leading to poor oxygenation. The resulting hypoxic environment recruits immune cells and further promotes tumor growth. Hypoxia, driven by the rapid growth of gliomas and inadequate vascularization, triggers the release of VEGF, which in turn exacerbates vascular permeability, inflammation, and edema, thereby perpetuating a detrimental feedback loop [76]. This cascade not only exacerbates tumor-associated edema but also affects the perivascular niche. Notably, endothelial nitric oxide (NO) signaling within the perivascular niche activates the Notch pathway, which promotes stem-like characteristics in glioma cells and advances tumor progression in PDGF-induced gliomas [77]. In alignment with these findings, Lathia et al. showed that the perivascular niche forms a supportive microenvironment for glioblastoma stem cells, with integrin α6 playing a crucial role in their adhesion and sustainment [78].

Spatial transcriptomic analysis has identified a subset of tumor-associated macrophages in the hypoxic regions of glioblastomas that compromise tumor vasculature via adrenomedullin secretion. In experimental GBM models, inhibition of adrenomedullin (ADM) normalized tumor vasculature, enhanced drug delivery, and showed therapeutic promise [79]. Similarly, vascular remodeling in glioblastomas can be influenced by matrix remodeling enzymes. Loss of MMP-2 activity in GBM results in hyperbranched but dysfunctional tumor vasculature, impairing blood perfusion and pericyte activation, subsequently prolonging survival in mouse models [80]. Integrating spatial transcriptomics and proteomics identified a five-layer spatial organization in glioblastoma driven by hypoxia gradients, which extends beyond histological observations of tumor architecture to include molecular details of cellular states [81].

## 5. Extracellular Matrix of the Glioma Microenvironment

The ECM comprises proteoglycans, fibrous proteins, and signaling molecules that provide structural support and regulate cellular behavior. Core components include collagen for strength, elastin for elasticity, fibronectin and laminins for adhesion, glycosaminoglycans for hydration, and enzymes such as matrix metalloproteinases (MMPs) and lysyl oxidases (LOX) for ECM remodeling and maintenance [82]. The brain’s extracellular matrix is distinct from other organs due to its high concentration of glycosaminoglycans (GAGs), such as hyaluronic acid (HA) and proteoglycans [83]. ECM-associated gene clusters, particularly those expressed by pericytes, are significantly elevated at recurrence and correlate with poorer survival outcomes [15].

NG2, a chondroitin sulfate proteoglycan, is implicated in GBM cell proliferation and EGFR signaling [84]. Notably, NG2-positive tumor cells demonstrate enhanced checkpoint activation and prolong survival subsequent to radiation exposure [85]. Peroxiredoxin-1 (PRDX-1) plays a pivotal role in mediating this resistance by promoting reactive oxygen species (ROS) scavenging and facilitating DNA damage repair [85]. Similarly, Perlecan/HSPG2 is markedly upregulated in glioblastoma, exhibiting a 13–14 fold increase [86]. Although glioblastoma cells with elevated heparan sulfate expression do not display increased proliferative activity, they contribute to tumor progression via ECM remodeling, thus fostering a more invasive tumor phenotype [86].

GBM cells exhibit distinct morphological and migratory behaviors within collagen-hyaluronan hydrogels; notably, higher concentrations of hyaluronic acid significantly restrict cell motility [87,88]. The interactions between CD44 and HA are essential for GBM invasion [88,89]. Furthermore, within HA-rich ECM, GBM cells employ CD44-dependent microtentacles (McTNs) for adhesion and migration, a process distinctly different from filopodia or pseudopodia migration mechanisms [89]. Accumulation of HA also serves as a physical barrier that limits drug penetration and reduces efficacy. Glioblastoma xenografts highly express hyaluronic acid, which impedes the spread of oncolytic viruses and diminishes therapeutic effectiveness [90]. To counteract this barrier, the hyaluronidase-expressing oncolytic adenovirus ICOVIR17 has been developed, which effectively degrades HA, enhances viral distribution, and promotes tumor regression [90,91]. In a murine GBM model, ICOVIR17-mediated HA degradation also facilitates NF-κB activation in macrophages, reversing the immunosuppressive tumor microenvironment and promoting anti-tumor immune responses [91].

Solid stress exerted by nodular brain tumors significantly reduces blood flow in the peritumoral vasculature, leading to hypoxia-induced neuronal apoptosis. This process contributes to neurological dysfunction, as demonstrated in both murine models and human patients [92]. Moreover, the HIF1α- tenascin C (TNC) feedback loop has been identified as a key regulator of ECM stiffness and glioma aggression, with mutant IDH1 suppressing HIF1α expression and reducing TNC deposition. Nevertheless, in recurrent tumors, this protective mechanism is circumvented, resulting in increased tumor stiffness and aggression [93].

## 6. Modifying the Microenvironment: Current Strategies and Future Directions

Comprehensive analysis of the brain TME suggests that modulating the diverse phenotypes of immune cells, rather than depleting them, could enhance the effectiveness of immunotherapies for brain cancers. Moreover, a deeper understanding of the immune landscape within the tumor microenvironment is crucial for the development of strategies to combat brain malignancies [27]. Kohanbash et al. found that mutations in IDH1 and IDH2 in gliomas suppress immune responses by reducing STAT1 expression and CXCL10 production, thereby decreasing CD8+ T cell infiltration [94]. Moreover, the study underscores that the use of the mutant IDH1 inhibitor IDH-C35 enhances the efficacy of peptide-based vaccines in a syngeneic mouse glioma model [94].

The CheckMate 143 trial revealed that although bevacizumab exhibited a higher objective response rate, nivolumab was associated with a prolonged duration of response, underscoring the potential role of immunotherapy in the treatment of glioblastoma [95]. Subsequent exploratory analyses suggested that the immune checkpoint inhibitor may offer substantial benefits, particularly in subgroups exhibiting MGMT promoter methylation [95]. Cloughesy et al. observed that neoadjuvant administration of pembrolizumab induced robust local and systemic immune responses, evidenced by enhanced T cell activity and increased expression of interferon-γ-related genes in glioblastoma tumors, highlighting the crucial timing of immunotherapy in treatment regimens [96]. Similarly, neoadjuvant nivolumab was found to augment T-cell diversity and immune infiltration in glioblastoma, indicative of enhanced immunomodulatory effects [97].

Cancer-associated fibroblasts in gliomas contribute to immune suppression by secreting collagen that interacts with the LAIR1 receptor, leading to T cell exhaustion [98]. Targeting the immune inhibitory receptor LAIR1 presents a novel therapeutic strategy for gliomas marked by collagen-rich microenvironments [98].

LOX influences macrophage infiltration through NF-κB-PATZ1 signaling, while CLOCK modulates microglial recruitment via the OLFML3 axis, both contributing to immune suppression in glioblastoma [99]. Notably, simultaneous targeting of LOX and CLOCK pathways effectively interrupted macrophage and microglial infiltration in PTEN-deficient glioblastoma, markedly enhancing anti-tumor immune responses [99]. Anti-CSF-1R therapy or the standard-of-care treatment induces fibrosis, which supports tumor cell survival and dormancy within fibrotic niches [100]. Given that TGF-β signaling and ECM remodeling are crucial in fibrosis-associated glioblastoma recurrence, strategies targeting fibrosis-related pathways have shown potential in enhancing the efficacy of anti-CSF-1R therapy and in reducing glioblastoma recurrence in preclinical models [100].

ERK1/2 phosphorylation (p-ERK) serves as a predictive biomarker for survival among recurrent glioblastoma patients undergoing PD-1 blockade [101]. Moreover, tumors exhibiting high-p-ERK levels display a distinct immune microenvironment enriched by myeloid cells and microglia expressing MHC class II, indicating a unique immunomodulatory landscape [101]. Glioblastoma-associated cranial bone niches harbor tumor-reactive CD8+ T cells that exhibit significant antitumor activity and resilience [102]. Additionally, emerging evidence supports CXCR4 radiolabeling as a potential biomarker for cranial immune responses, which correlates with improved patient survival [102].

Zman-seq, a time-resolved single-cell RNA sequencing technique, traced the timeline of immune cell state trajectories and identified TGFβ as a critical regulator of NK cell dysfunction [42]. Furthermore, preclinical studies have shown that combining CSF1R and PD1 inhibition effectively counteracts the immunosuppressive tumor milieu in H3-mutant glioma [103]. In addition to immune modulation, repurposed neuroactive drugs, such as vortioxetine, have demonstrated potent anti-glioblastoma effects through activation of the AP-1/BTG tumor suppressor pathways [104]. Mechanistically, calcium-mediated signaling and AP-1 activation are crucial in driving glioblastoma cell apoptosis and reducing tumor growth in preclinical models [104]. In neuron-GBM interactions, the engineered peptide K90-114TAT disrupts the EAG2–Kvβ2 potassium channel complex, effectively attenuating tumor growth and invasion by targeting a key pathway in glioblastoma progression [105].

Future therapeutic approaches for glioma treatment will focus on personalized and combinatorial strategies that integrate immunotherapy, metabolic modulation, and microenvironment-targeting agents [8]. Advances in single-cell and spatial transcriptomics will further elucidate glioma heterogeneity, aiding in the identification of novel therapeutic targets [106]. Emerging modalities such as engineered immune cells, oncolytic viruses, and nanotechnology-based drug delivery hold promise for overcoming glioma’s resistance mechanisms.

## 7. Conclusions

Gliomas remain one of the most challenging malignancies due to their complex and adaptive tumor microenvironment. The progression of glioma is profoundly influenced by the TME, and modifying its functions can significantly restrain tumor growth. Despite advances, the treatment options for glioma are still limited, and clinical translation faces significant challenges due to model limitations, tumor heterogeneity, the blood-brain barrier, and immunosuppression. Substantial progress has been achieved in elucidating the complexities of tumor-microenvironment interactions. Future research needs to focus on refining experimental models, incorporating patient-derived organoids, and leveraging advanced imaging and omics technologies. Integrating multimodal strategies that combine immune modulation, metabolic targeting, and enhanced drug delivery across the blood-brain barrier will be essential for developing more effective therapies and improving patient outcomes.
