# Correction: Jang, H.J.; Park, J.-W. Microenvironmental Drivers of Glioma Progression. *Int. J. Mol. Sci.* 2025, *26*, 2108

**DOI:** 10.3390/ijms27031335

**Published:** 2026-01-29

**Authors:** Hyun Ji Jang, Jong-Whi Park

**Affiliations:** 1 Department of Life Sciences, College of BioNano Technology, Gachon University, Seongnam 13120, Republic of Korea; 2 Department of Health Sciences and Technology, GAIHST, Gachon University, Incheon 21999, Republic of Korea

After the publication of this review article [1], it was identified that one of the referenced manuscripts had been retracted. As no suitable replacement reference was available, the authors decided to remove the previous reference number 99 [2] and its related in-text citations from the manuscript.

With this correction, the order of some references has been adjusted accordingly. This correction does not affect the scientific conclusions of the article. This correction was approved by the Academic Editor. The original publication has also been updated.
